# Effect of fatigue caused by a simulated handball game on ball throwing velocity, shoulder muscle strength and balance ratio: a prospective study

**DOI:** 10.1186/s13102-016-0038-9

**Published:** 2016-05-05

**Authors:** Marília Santos Andrade, Fabiana de Carvalho Koffes, Ana Amélia Benedito-Silva, Antonio Carlos da Silva, Claudio Andre Barbosa de Lira

**Affiliations:** Departamento de Fisiologia, Universidade Federal de São Paulo (UNIFESP), Rua Botucatu, 862, 5º andar, Vila Clementino, São Paulo, SP CEP: 04023-062 Brazil; Escola de Artes, Ciências e Humanidades, Universidade de São Paulo (USP), Av. Arlindo Béttio, 1000, Ermelino Matarazzo, São Paulo, SP CEP: 03828-000 Brazil; Setor de Fisiologia Humana e do Exercício, Faculdade de Educação Física e Dança, Universidade Federal de Goiás (UFG), Avenida Esperança s/n, Campus Samambaia, Goiânia, GO CEP: 74690-900 Brazil

**Keywords:** Isokinetic dynamometer, Handball, Shoulder strength, Ball throwing velocity

## Abstract

**Background:**

Arm throwing represents a deciding element in handball. Ball velocity, aim accuracy, and dynamic stability of the shoulder are factors that influence throwing effectiveness. The purpose of this study was to examine the influence of muscle fatigue caused by simulated game activities (SGA) on shoulder rotational isokinetic muscle strength, muscle balance and throwing performance, and to examine the relationship between muscle strength and throwing performance.

**Methods:**

Ten national elite adult handball athletes were evaluated. Isokinetic internal (IR), external (ER) rotators peak torque, and balance ratio were measured before and after SGA. Ball throwing velocity was assessed by radar gun.

**Results:**

Both internal (IR) and external (ER) rotators peak torque were significantly lower after SGA (*p* = 0.0003 and *p* = 0.02, respectively). However, the deleterious effect was more evident for IR than ER muscles (effect size *r* = 0.39 and *r* = 0.18, respectively). Balance ratio before and after SGA did not differ (*p* = 0.06). Ball throwing velocity was not impaired by SGA. Moreover, isokinetic variables correlated positively with ball velocity (*r* ≥ 0.67).

**Conclusions:**

SGA affected the muscle strength of IR more than ER, predisposing the shoulder joint to muscular imbalance. The muscular impairment after SGA was insufficient to impair ball throwing velocity.

**Electronic supplementary material:**

The online version of this article (doi:10.1186/s13102-016-0038-9) contains supplementary material, which is available to authorized users.

## Background

Throwing velocity in overarm throwing is crucial in sports such as baseball, handball, javelin, volleyball, and water polo [1]. Glenohumeral stabilization and muscle strength are among the most important factors for developing ball velocity [2, 3], and several authors have studied the association of this factor [4–6]. Joint stabilization and muscular strength are also of fundamental importance to prevent joint injury [7–9]. With regard to this strength component, recent findings have shown that some overhead athletes have a strength imbalance between internal and external rotator shoulder muscles [4, 10–12] and consequently exhibit poor joint stabilization. In order to reduce this strength imbalance, core stability exercises have been suggested [13].

Another interesting finding drawn from the literature is that there is a higher incidence of injuries late in the game *vs* early in the game [14]. Thus, the muscular fatigue caused by repetitive movement during a game may be more evident in the agonist throwing muscles than in the antagonist muscles. This selective fatigue can affect muscular strength balance and consequently shoulder joint stabilization, which may be a factor contributing to the higher injury incidence late in the game. A greater involvement of elastic tissues in the presence of muscular fatigue, reducing the need for muscle activation, may also be associated with joint injury [15]. In addition, this fatigue may represent a factor impairing ball throwing performance, as previously demonstrated for basketball players [16].

Traditionally, muscle performance is assessed by isokinetic dynamometer, however adequate patient stabilization and clear instructions during the test are fundamental for good quality and reproducible data [4, 17]. This device provides coaches, clinicians, and physical therapists an objective evaluation of muscle strength and strength balance ratio between shoulder external (ER) and internal rotators (IR) muscles [4, 11, 12].

Although many authors agree on the importance of muscle strength for arm throwing, only limited conclusions can be drawn about the relationship between upper limb strength and throwing performance because results available are conflicting [18–21].

Therefore, we hypothesized that muscular strength, muscular balance between internal and external rotator muscles and ball throwing velocity are affected by the muscular fatigue caused by a game, or in the present case by a simulated game involving several muscle actions present in a real game. Moreover, we also hypothesized that there is a significant relationship between isokinetic muscle strength and ball throwing velocity.

Therefore, the purpose of this study was: i) to examine the influence of muscular fatigue caused by simulated game activities (SGA) on isokinetic muscle strength, balance ratio and ball throwing velocity; and ii) to examine the relationship between shoulder rotational strength and ball velocity in handball athletes.

## Methods

### Participants

Participants in this study comprised a convenience sample of 10 highly trained, competitive, professional male handball players from Brazil. The players had a mean age of 23.1 ± 2.8 years (range: 20–29 years), mean height of 185.0 ± 7.7 cm (range: 172–193 cm), and mean body mass of 87.1 ± 11.3 kg (range: 66–105 kg). All participants were recruited from an elite team that participates in national and international championships and had engaged in handball training for a mean of 10.2 ± 4.0 years (range: 5–18 years). Exclusion criteria were shoulder pain or injury within the year leading up to the study. Four athletes had shoulder injuries predating this time period.

Participants were informed of the potential risks and benefits of the study and signed an informed consent form to take part. All experimental procedures were approved by the Federal University of São Paulo Human Research Ethics Committee and conformed to the principles outlined in the Declaration of Helsinki.

### Study design

Upon arrival at the laboratory, participants underwent isokinetic evaluation of their dominant upper limb. Tests began at 2 pm for all the athletes in order to avoid the influence of the circadian rhythm on muscle strength [22]. Limb dominance was determined by identifying the upper limb that the participant prefers to use to throw a ball [23]. Ten minutes after isokinetic evaluation, the participants performed eight standing and eight jumping arm throws on the handball court to measure ball velocity.

Immediately after completion of the throwing activities, athletes performed a program of simulated game activities (SGA) to simulate the upper limb muscular stress of a real handball game. Five minutes after the end of the SGA, arm throwing action was repeated to measure ball velocity . Finally, the athletes were submitted to another isokinetic strength test. All the evaluation tests were done in this sequence and on the same day.

### Isokinetic assessment

Before isokinetic testing, a 5-min warm-up was performed on an arm-cycle ergometer (Cybex Inc., Ronkonkoma, NY, USA) at a resistance level of 25 W, and standardized static stretching exercises were performed, since it has been demonstrated that these static exercises in association with warm-up exercises performed prior to isokinetic strength tests have no influence on strength results [24, 25]. To stretch the glenohumeral joint muscles, the following exercises were performed in this order: arm abduction at 90 deg with horizontal flexion targeting the posterior deltoid fibers; shoulder abduction with the upper limb behind the head targeting the triceps muscle; upper limb abduction to 135 deg with horizontal extension targeting the pectoralis major muscle; upper limb abduction at 90 deg with horizontal extension targeting the pectoralis minor muscle; and shoulder internal rotation with the hand behind the body targeting the external rotator muscles. All the static stretching exercises were performed in two unassisted successive repetitions for 15-s up to a threshold of mild discomfort, with no pain acknowledged by the athletes. Between each stretching repetition, during stretching exercises and at each muscle group change, the upper limb was rested for a 15-s period in a neutral position. Following the stretching period, participants were placed in the isokinetic dynamometer (Biodex Medical Systems Inc., Shirley, NY, USA) to evaluate maximal strength during positive (concentric) and negative (eccentric) exercises for the dominant limb [26].

The IR and ER muscles were assessed in the seated position, with the upper limb abducted at 90 deg on the frontal plane and the elbow flexed at 90 deg. The range of motion reached 50 deg for internal rotation and 70 deg for external rotation. The isokinetic velocities selected were 60 deg∙s^−1^ and 300 deg∙s^−1^ in the concentric mode (positive exercise) and 90 deg∙s^−1^ and 300 deg∙s^−1^ in the eccentric mode (negative exercise). Participants performed three submaximal trials to familiarize themselves with the range of motion and the accommodating resistance of the dynamometer. Participants then performed a maximum of five repetitions to test each velocity used. Positive exercise tests were done first; lower test velocities were performed before faster velocities. Peak torque, total work, and conventional strength ratio (calculated as positive external rotation-to-positive internal rotation ratio) was assessed at 60 deg∙s^−1^, peak torque and average power was assessed at 300 deg∙s^−1^, and functional strength ratio was calculated as negative external rotator peak torque at 90 deg∙s^−1^ to positive internal rotator peak torque at 300 deg∙s^−1^. Successive velocity testing was separated by one-minute rest intervals.

Before testing, the dynamometer was calibrated according to the manufacturer’s specifications and checked prior to testing each participant. Standardized verbal encouragement was given during all testing. Visual feedback from the computer screen was not allowed.

### Throwing performance

Ball velocity was measured by a radar gun (Stalker Sport, Stalker Radar, Texas, USA) according to Cools et al. [27]. To this end, participants performed two types of throw: one was in a standing position seven meters from the goal; the second (from the nine meter line) was a jumping throw preceded by two steps. All participants threw the ball in this order, and performed eight arm throws in a standing position and eight in a jumping position, two into each corner of the goal. Ball velocity was measured for all arm throws, where the velocity used was the mean of the eight throws. This test was performed on an official handball court.

To establish test-retest reliability, subjects were invited to participate in a second measurement session at 3–4 days after the initial assessment, during which ball velocity on both tests was assessed in the same way as in the first session. A time interval of 3–4 days was chosen to avoid the training effect.

### Simulated game activities (SGA)

Simulated game activities were designed to simulate the upper limb muscular stress of a real handball game. The SGA were based on the mean number of steps and throws toward goal registered for each team player position in the last three games. Therefore, the exercise protocol devised included 100 steps and 20 arm throws at goal.

During the SGA, the heart rate (HR) of all participants was monitored using a heart rate monitor (Suunto Team Pod, Suunto Oy, Vantaa, Finland) with the purpose of monitoring exercise intensity. The mean HR during SGA was 153 ± 13 bpm, which represents approximately 77 % of the maximal predicted HR (220-age) [28] for the group of handball players.

### Statistical analysis

All variables presented normal distributions according to the Shapiro-Wilk test. In the pre-SGA condition, the association between ball velocity and isokinetic muscular performance was evaluated by calculating Pearson’s correlation coefficients (r) and classifying according to the following rule: no correlation for *r* < 0.50; moderate for 0.50 ≤ *r* < 0.75; and good-to-strong for *r* ≥ 0.75 [29].

Paired *t*-tests were used to compare the effect of SGA on isokinetic muscular strength and ball velocity. The significance level (α) was set at 0.05 for all statistical procedures. The results were also assessed for clinical significance by using effect size (ES) of changes. ES was calculated and classified as follows: large for ES ≥ 0.8, moderate for 0.5 ≤ ES < 0.8, small for 0.2 ≤ ES < 0.5 and trivial for ES < 0.2 [30]. Confidence intervals (90 %) were also calculated. All statistical analyses were performed with Statistica version 7.0 software (Statsoft Inc., Oklahoma, USA).

An intraclass correlation coefficient (ICC) was used to assess test-retest reliability of both ball velocity tests (jumping or in standing position). ICC values of less than 0.40 were considered poor, 0.40–0.59 fair, 0.60–0.74 good, and .075–1.0 excellent [31].

## Results

Analysis of the ICC for the ball velocity test in a standing position, the value was classified as good (0.71) while in the jumping throw preceded by two steps, ICC was classified as excellent (0.99).

Table 1 depicts the mean and standard deviations of shoulder ER and IR positive peak torque and total work (at 60 deg∙s^−1^), positive peak torque and mean power (at 300 deg∙s^−1^), and negative peak torque (at 90 deg∙s^−1^ and 300 deg∙s^−1^), as well as balance ratio (conventional and functional strength ratios). Values shown represent before and after SGA.

**Table 1:** Effects of simulated game activities on isokinetic parameters for dominant limbs of handball athletes

	Before	After	*P* value	ES	IC
External rotator					
Positive peak torque at 60 deg.s^−1^ (Nm)	43.3 ± 7.7*	40.6 ± 6.4	0.02	0.38	−0.13 to −0.63
Positive total work at 60 deg.s^−1^ (J)	51.7 ± 9.8*	46.7 ± 8.5	0.01	0.54	−0.24 to-0.84
Positive peak torque at 300 deg.s^−1^ (Nm)	35.2 ± 5.0	34.5 ± 2.5	0.41	0.17	0.19 to −0.53
Average power at 300 deg.s^−1^ (W)	65.3 ± 14.4	59.2 ± 10.3	0.12	0.48	0.032 to −0.99
Negative peak torque at 90 deg.s^−1^ (Nm)	45.2 ± 9.8	38.9 ± 9.5	0.06	0.65	−0.096 to −1.2
Negative peak torque at 300 deg.s^−1^ (Nm)	44.7 ± 6.2*	40.4 ± 4.5	0.04	0.79	
Internal rotator					
Positive peak torque at 60 deg.s^−1^ (Nm)	71.3 ± 13.7*	60.9 ± 10.2	0.01	0.86	−0.37 to −1.3
Positive total work at 60 deg.s^−1^ (J)	90.9 ± 16.9*	77.8 ± 13.1	0.00	0.86	−0.86 to −0.86
Positive peak torque at 300 deg.s^−1^ (Nm)	66.5 ± 12.3*	61.3 ± 12.3	0.00	0.42	−0.42 to −0.42
Average power at 300 deg.s^−1^ (W)	113.3 ± 29.4*	94.8 ± 29.1	0.02	0.63	−0.22 to −1
Negative peak torque at 90 deg.s^−1^ (Nm)	79.8 ± 13.6	68.9 ± 17.2	0.06	0.70	−0.1 to −1.3
Negative peak torque at 300 deg.s^−1^ (Nm)	69.9 ± 14.6	62.9 ± 12.9	0.16	0.51	0.051 to −1.1
Balance ratios					
Conventional ratio	0.61 ± 0.1	0.67 ± 0.1	0.063	0.60	−0.081 to −1.1
Functional ratio	0.68 ± 0.1	0.67 ± 0.2	0.806	0.06	0.37 to −0.49

Data are expressed as mean ± standard deviation. *ES* effect size, *IC* interval confidence

*p < 0.05, differs from after, Student *t* test

Student’s *t*-test for dependent samples showed significantly lower values of peak torque and total work (at 60 deg∙s^−1^) for ER and IR muscles after SGA. Effect size of SGA on IR muscles was higher for IR muscles than for ER muscles across all isokinetic variables.

Conventional strength ratios ranged from 0.61 ± 0.10 to 0.67 ± 0.10 at 60 deg.s^−1^ (*p* = 0.06) and functional strength ratios from 0.68 ± 0.10 to 0.67 ± 0.20 at 300 deg∙s^−1^ (*p* = 0.81). Changes in conventional ratio after SGA had moderate ES.

In a subset analysis, the four athletes reporting history of injury predating the year before the study had a functional strength ratio of 0.62 ± 0.10, whereas the remaining 6 players had a functional ratio of 0.75 ± 0.10. The conventional strength ratio for the four previously injured athletes was 0.58 ± 0.12 and for the remaining six athletes was 0.63 ± 0.14. There was no significant difference between the injured and uninjured groups.

Table 2 shows Pearson’s correlation coefficients for isokinetic and throwing performance (*r*-values ranged from 0.67 to 0.82). Concerning ER muscles, a negative, moderate and significant correlation with ball velocity on the standing arm throw was found for functional ratio (*r* = −0.65, *p* = 0.043).

**Table 2:** Correlation coefficients between isokinetic parameters and ball velocity during standing or jumping arm throwing in male handball players

		Standing arm throwing		Jumping arm throwing	
		*R* value	*P* value	*R* value	*P* value
Positive peak torque at 60 deg.s^−1^	External rotator	0.61	0.060	0.77	0.009
	Internal rotator	0.82	0.004	0.89	0.001
Positive total work at 60 deg.s^−1^	External rotator	0.62	0.058	0.71	0.020
	Internal rotator	0.79	0.006	0.82	0.004
Positive peak torque at 300 deg.s^−1^	External rotator	0.68	0.029	0.75	0.012
	Internal rotator	0.83	0.003	0.86	0.001
Average power at 300 deg.s^−1^	External rotator	0.63	0.052	0.60	0.065
	Internal rotator	0.62	0.058	0.62	0.054
Negative peak torque at 90 deg.s-1	External rotator	0.53	0.118	0.67	0.036
	Internal rotator	0.67	0.022	0.73	0.031
Negative peak torque at 300 deg.s^−1^	External rotator	0.29	0.424	0.40	0.258
	Internal rotator	0.71	0.022	0.68	0.031
	Conventional ratio	−0.23	0.525	0.12	0.732
	Functional ratio	−0.65	0.043	0.57	0.081

Variability of ball velocity among handball players was high at almost 30 %. Student’s *t*-test for dependent samples showed no significant differences in ball velocity for throwing in jumping or standing positions before and after SGA (*p* > 0.05) (Table 3). The statistical power for these analyses ranged from 5.6 to 14.4 %.

**Table 3:** Throwing performance before and after simulated game activities

	Simulated game activities		
	Before	After	*P* value
Ball velocity - standing throwing (m.s^−1^)	23.6 ± 3.1	23.2 ± 3.2	0.09
Ball velocity - jumping throwing (m.s^−1^)	22.8 ± 2.5	22.5 ± 3.2	0.23

Data are expressed as mean ± standard deviation

## Discussion

The primary purpose of this study was to ascertain whether SGA could affect muscular strength and balance as well as ball velocity in male handball players. The study also sought to determine whether isokinetic muscle variables were associated with ball throwing velocity in male handball players. To our knowledge, this is the first paper to demonstrate the effect of SGA on a functional task (ball velocity) and on isokinetic parameters in male handball players. The findings reported lead us to conclude that SGA causes a decrease in muscle strength and probably induces muscular imbalance, without significant changes in ball throwing velocity. Moreover, the data also showed a significant correlation between isokinetic muscle strength and ball throwing velocity. Statistical significance and ES were employed to interpret the data because it allows researchers to move away from the simple identification of statistical significance toward a more generally interpretable, quantitative description of an effect, independent of the potentially misleading influence of sample size.

Isokinetic variables of the positive IR test were reduced after SGA for all variables evaluated but this strength reduction was greater in IR than in ER. The muscle strength balance ratio tended to be affected by SGA (*p* = 0.06) and the ES was moderate. In this context, the higher incidence of injuries late in the game *vs* early in the game, suggested by Hawkins and Fuller [14], may be related to a loss of muscular balance due to the greater muscular fatigue in IR than in ER muscles caused by SGA. Therefore, it is likely that rehabilitation or injury prevention programs aimed at improving IR muscles endurance may be helpful to avoid muscular strength loss and consequently muscular joint imbalance at end of games.

Zapartidis et al. [32] examined the influence of SGA on throwing effectiveness and rotational strength of the shoulder. They found that SGA did not impair rotational strength of the shoulder except in the case of ER at 180 deg∙s^−1^. One possible explanation for the discrepancy between the present study results and those of Zapartidis et al. [32] is that our volunteers were men whereas the cited authors’ were women. Fatigue caused by the game might produce different levels of impairment in muscle performance in men and women. Future studies, including muscle endurance evaluation, are necessary to elucidate this question.

The mean throwing velocity achieved by athletes in the present study before SGA was 23.6 m∙s^−1^ (18.1–27.1 m∙s^−1^). These results were higher than values reported by Jöris et al. [2] and Zapartidis et al. [32], a difference likely explained by the fact that the subjects in the previous studies were female handball players, where higher ball velocities are to be expected among male athletes.

A plethora of studies have investigated muscle strength and balance of the shoulder muscles in an attempt to identify a relationship with throwing arm velocity [18, 20, 33]. It is reasonable to presume a relationship between ball velocity and strength developed by the throwing arm, but a review of other sports activities described in the literature has shown that the relationship between isokinetic results and field performance varies widely [18, 20, 33].

Our results showed a strong relationship between ER and IR peak torque at 60 and 300 deg∙s^−1^, total work at 60 deg∙s^−1^, and ball velocity in both jumping and standing throws. One finding is that IR shoulder strength almost always correlates more strongly with ball velocity than ER shoulder strength. This is expected because IR muscles are responsible for accelerating the limb during the throwing action.

It is important to emphasize that related findings do not necessarily prove cause and effect between muscle strength and ball velocity. Since throwing is a multi-joint action, a variety of factors may contribute to this movement. Indeed, Atwater [34] showed throwing to be a complex motion involving all body parts. It has been suggested that approximately 50 % of ball throwing velocity is the result of body rotation, while the remainder is the result of upper-extremity action. Further, Pappas et al. [35] have described the anatomical sequence of throwing as proceeding from the fixed foot, up through the pelvis and trunk, to the upper extremity and therefore the importance of the lower extremity movement and trunk rotation should not be underestimated when examining throwing movements. Thus, high ball velocity is not produced solely by greater shoulder muscle strength, but athletes who have strong shoulder muscles may also have greater strength in their lower limbs, pelvis and trunk musculature. This implies that their ball velocity test would be higher as a consequence of the greater strength in their entire body. The findings presented in this study lead us to postulate that improvement of IR and ER strength, whether at low or high velocities, may increase ball velocity during throwing. However, for the reasons given above this conclusion should be interpreted with caution.

Interestingly, ball velocity showed a significant negative relationship with functional strength ratio at 300 deg∙s^−1^. Forthomme et al. [19] also showed a significant negative relationship between the functional strength ratio at 400 deg∙s^−1^ and spike velocity in volleyball players. These findings may suggest that lower strength ratios are beneficial for high arm throwing velocity. However, it is well known that shoulder stability is largely maintained by the ligaments and musculotendinous units [36] and sports such as handball that involve repetitive overarm motion require shoulder stability with well-coordinated and synchronized actions of shoulder muscles to prevent injury in this joint [9, 18, 19]. Several authors agree that in an overhead throwing athlete, an adequate ratio of negative antagonist muscle strength to positive agonist muscle strength is critical for dynamic stability and optimal function [9, 36]. In the present study, the four players reporting a history of shoulder tendinosis or superior labrum from anterior to posterior lesion, showed a tendency towards lower functional strength ratios. There was no significant difference between the groups but since the sample size for this analysis was very small, statistical analysis of this data should be analyzed cautiously. The four players who had previous injuries exhibited a mean functional strength ratio of 0.62 ± 0.10 whereas the remaining six uninjured players presented a mean functional ratio of 0.75 ± 0.10. The conventional strength ratio also showed a tendency to be lower among athletes who had a history of shoulder lesion than for those who presented no lesion (0.58 ± 0.12 and 0.63 ± 0.14, respectively). Therefore, although IR strengthening and low functional strength ratio seems to be beneficial for enhanced ball throwing velocity, we advise against shoulder strength imbalance because of the increased risk of injuries [10, 37].

To our knowledge, this paper is the first which demonstrate the effect of SGA on ball velocity and isokinetic strength parameters in male handball players. Another strength is the use of statistical significance and ES in data analysis, allow researchers to move away from the simple identification of statistical significance toward a more generally interpretable, quantitative description of an effect, independent of the possible misleading influence of sample size. However, data after a real handball game was not analyzed where SGA data was used instead, representing a possible limitation of the study. Further studies verifying the effects of a real handball game on shoulder muscular strength balance should be carried out. Studies investigating whether there is a causal effect between shoulder muscular balance and ball velocity should also be conducted.

## Conclusion

SGA had a greater effect on IR than ER muscle strength, likely creating a muscle imbalance in the shoulder joint, which may predispose this to a higher injury risk. These findings may be helpful to exercise and sports science professionals since improving IR muscles endurance may prevent muscular imbalance at end of games. Despite this muscle strength reduction, ball velocity was not affected. Functional strength ratio had a negative correlation with ball velocity. However, muscular imbalance should be discouraged because of the increased risk of injury.

### Ethics approval

Participants were informed of the potential risks and benefits of the study and signed an informed consent form to take part. All experimental procedures were approved by the Federal University of São Paulo Human Research Ethics Committee and conformed to the principles outlined in the Declaration of Helsinki.

### Availability of data and materials

The dataset supporting the conclusions of this article is included in additional file [see Additional file 1].

## Additional file

Additional file 1: Availability of raw data. (XLS 25 kb)

## Abbreviations

ER: External rotators
ES: Effect size
HR: Heart rate
IR: Internal rotators
r: Pearson’s correlation coefficient
SGA: Simulated game activities
